# Formation and stability of complex metallic phases including quasicrystals explored through combinatorial methods

**DOI:** 10.1038/s41598-019-43666-w

**Published:** 2019-05-09

**Authors:** Witor Wolf, Sebastian A. Kube, Sungwoo Sohn, Yujun Xie, Judy J. Cha, B. Ellen Scanley, Claudio S. Kiminami, Claudemiro Bolfarini, Walter J. Botta, Jan Schroers

**Affiliations:** 1 Programa de Pós-Graduação em Ciência e Engenharia de Materiais, Universidade Federal de São Carlos, Rod. Washington Luiz, Km 235, São Carlos, SP 13565-905 Brazil; 2 Departamento de Engenharia Metalúrgica e de Materiais, Universidade Federal de Minas Gerais, Av. Antônio Carlos, 6627, Belo Horizonte, MG 31270-901 Brazil; 3 Department of Mechanical Engineering and Materials Science, Yale University, New Haven, Connecticut 06511 USA; 4 Department of Physics, Southern Connecticut State University, New Haven, Connecticut 06515 USA; 5 Departamento de Engenharia de Materiais, Universidade Federal de São Carlos, Rod.Washington Luiz, Km 235, São Carlos, SP 13565-905 Brazil

**Keywords:** Design, synthesis and processing, Characterization and analytical techniques

## Abstract

Aluminum-based quasicrystals typically form across narrow composition ranges within binary to quaternary alloys, which makes their fabrication and characterization challenging. Here, we use combinatorial approaches together with fast characterization techniques to study a wide compositional range including known quasicrystal forming compositions. Specifically, we use magnetron co-sputtering to fabricate libraries of ~140 Al-Cu-Fe and ~300 Al-Cu-Fe-Cr alloys. The alloys compositions are measured through automated energy dispersive X-ray spectroscopy. Phase formation and thermal stability are investigated for different thermal processing conditions (as-sputtered and annealed at 400 °C, 520 °C and 600 °C for Al-Cu-Fe libraries; annealed at 600 °C for Al-Cu-Fe-Cr libraries) using automated X-ray diffraction and transmission electron microscopy. In both systems the compositional regions across which the quasicrystalline phase forms are identified. In particular, we demonstrate that the quasicrystalline phase forms across an unusually broad composition range in the Al-Cu-Fe-Cr system. Additionally, some of the considered alloys vitrify during sputtering, which also allows us to study their nucleation behavior. We find that phases with polytetrahedral symmetry, such as the icosahedral quasicrystal and the λ-Al_13_Fe_4_ phase, exhibit higher nucleation rates but lower growth rates, as compared to other phases with a lower degree of polytetrahedral order. Altogether, the here used combinatorial approach is powerful to identify compositional regions of quasicrystals.

## Introduction

Since the discovery of quasicrystals (QCs) by D. Shechtman *et al*.^1^, more than a hundred metallic systems were found to form quasicrystalline phases such as Al-Cu-Fe^2–4^, Al-Cu-Fe-Cr^5^, Al-Fe-Cr-(TM = transition metal)^6–8^, Al-Mn-(TM)^9,10^, Al-Ni-Fe^11^ and Al-Ni-Co^12,13^. As the formation motifs of QCs are to a large extent due to geometry, size differences and size ratio of constitutive elements, QCs usually form in narrow composition ranges^13^, and are often metastable^7^. Phases with a similar atomic arrangement as QCs, so called approximants, can often be found in their vicinity^14,15^.

The study of composition ranges of QC formation is usually conducted using conventional metallurgy. This approach is challenging for multi-component alloys due to its slow rate of exploring new compositions: The potential QC composition space is large, but composition ranges for QC formation are rather narrow and phase transitions can often occur within variations of only 1 at.%. Thus, discovering new QC compositions and identifying their stability ranges has proven challenging and time consuming. One strategy to deal with complex multicomponent alloys in materials science and metallurgy uses combinatorial synthesis coupled with high-throughput analysis. Such approaches have been successfully applied to the study of metallic glasses^16–19^, high entropy alloys^20–22^, shape memory alloys^23,24^ and microstructural evolution^25–27^.

In this work, we use a combinatorial strategy to fabricate and characterize large composition ranges of the quasicrystal-forming systems, Al-Cu-Fe and Al-Cu-Fe-Cr. We use magnetron co-sputtering to fabricate compositional “alloy libraries” and we structurally evaluate such libraries in the as-sputtered state (far away from equilibrium) and in annealed states at different temperatures to approach equilibrium conditions. Using this approach, the QC formation range can be identified, suggesting this method as an effective tool for discovering potential QC compositions in large compositional regions. Although QC phases forming in the Al-Cu-Fe-Cr system have been previously reported^28–35^, the complete range of QC formation is unknown to date and will be elucidated by the here presented results. In addition, the results obtained in this work can be used for predicting intermetallic phase stability of Al-Cu-Fe sputtered films over a wide compositional and temperature range. Over a large portion of the Al-Cu-Fe as-sputtered library amorphous structures were obtained. Closer investigation reveals that phases with polytetrahedral symmetry, such as quasicrystal and approximants exhibit a more refined grain structure after thermal treatments, which suggests that nucleation rates are higher here than in other phases present. We interpret, as has been suggested previously^36–38^, the high nucleation rate of polyhedral phases as an indication of their structural similarity to the liquid/amorphous structure. This similarity is thought to lower the activation barrier for nucleation and hence lead to higher nucleation rates. However, aspects of nucleation, which are associated with required chemical fluctuations, as opposed to topological fluctuations, are challenging to quantify theoretically^39^. Using the method proposed here we can observe nucleation and growth of complex phases from an amorphous phase, which is structurally similar to the liquid phase across a wide composition range, which contributes to advancing nucleation theory to include both, chemical and topological fluctuations.

## Results and Discussion

### Preparation of Al-Cu-Fe libraries

**Figure 1:**
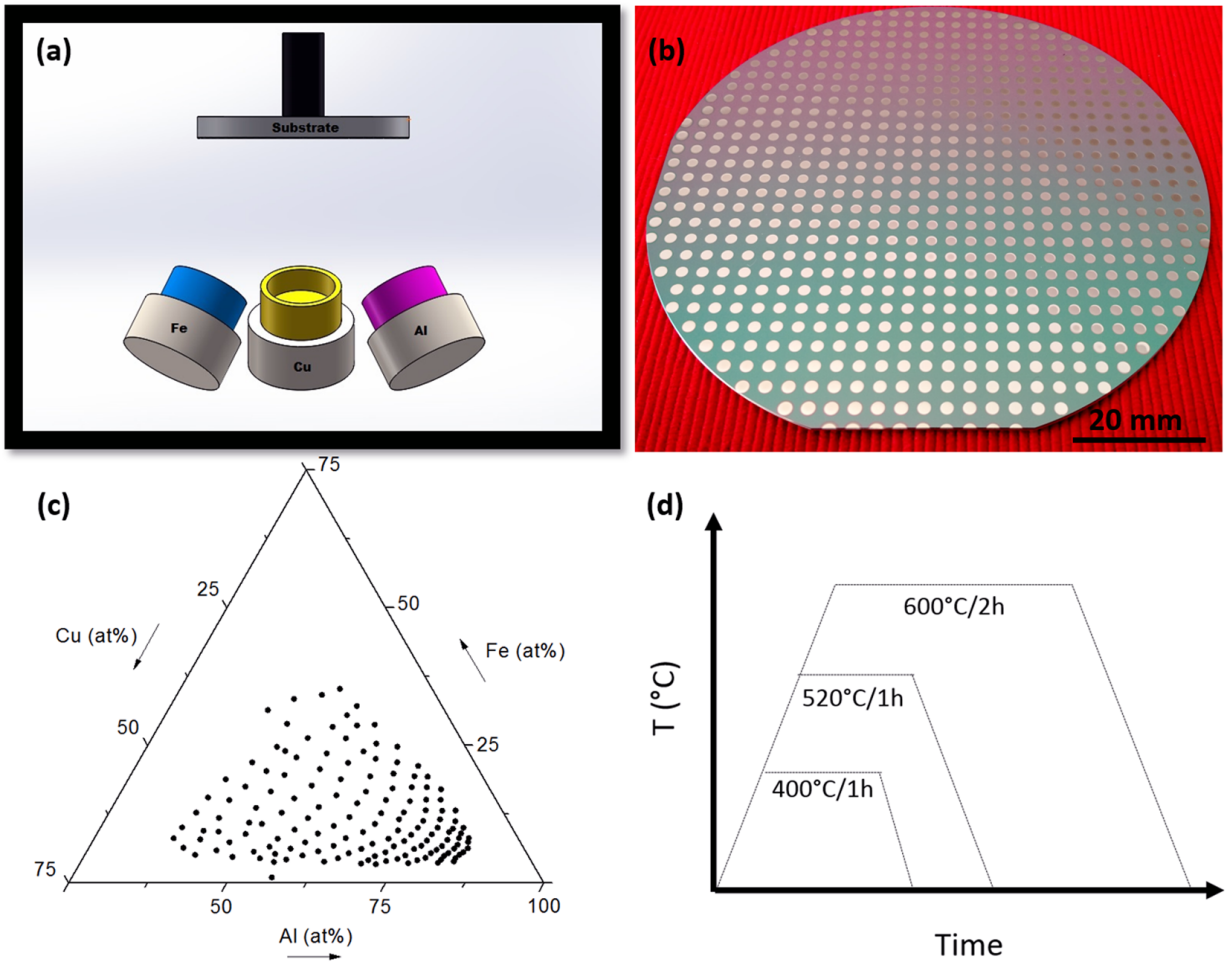
Compositional library fabrication and annealing. (**a**) Schematic of the DC magnetron co-sputtering setup used to synthesize the compositional libraries. This sputtering system is equipped with three targets of 50 mm diameter arranged in a tetrahedral configuration. The 100 mm substrate is placed on the opposite side of the targets. (**b**) Al-Cu-Fe compositional library. In total 147 alloys were evaluated. Every alloy has a defined position “xy” on the wafer so it is possible to trace every characterization made to a specific composition (alloy/patch). (**c**) High-throughput compositional mapping done by an automated EDX, plotted on a Gibbs triangle. (**d**) Annealing steps to study phase stability in different temperatures. Annealing was carried out at 400 °C, 520 °C and 600 °C.

Al-Cu-Fe libraries were fabricated by magnetron co-sputtering of elemental targets followed by annealing at 400 °C, 520 °C and 600 °C for 1 h, 1 h and 2 h respectively (Fig. 1). Each library (Fig. 1b) consists of individual metallic films (1.8 mm patches) on a silicon wafer with corresponding xy-coordinates, which allow for controlled measurements of chemical composition and subsequent structural characterization. The as-sputtered libraries were characterized using automated energy dispersive X-ray spectroscopy (EDX) to determine the composition gradient obtained in the co-sputtering process (Fig. 1c). Overall a total of 147 alloys were fabricated and characterized per library.

### Phase identification in Al-Cu-Fe libraries

**Figure 2:**
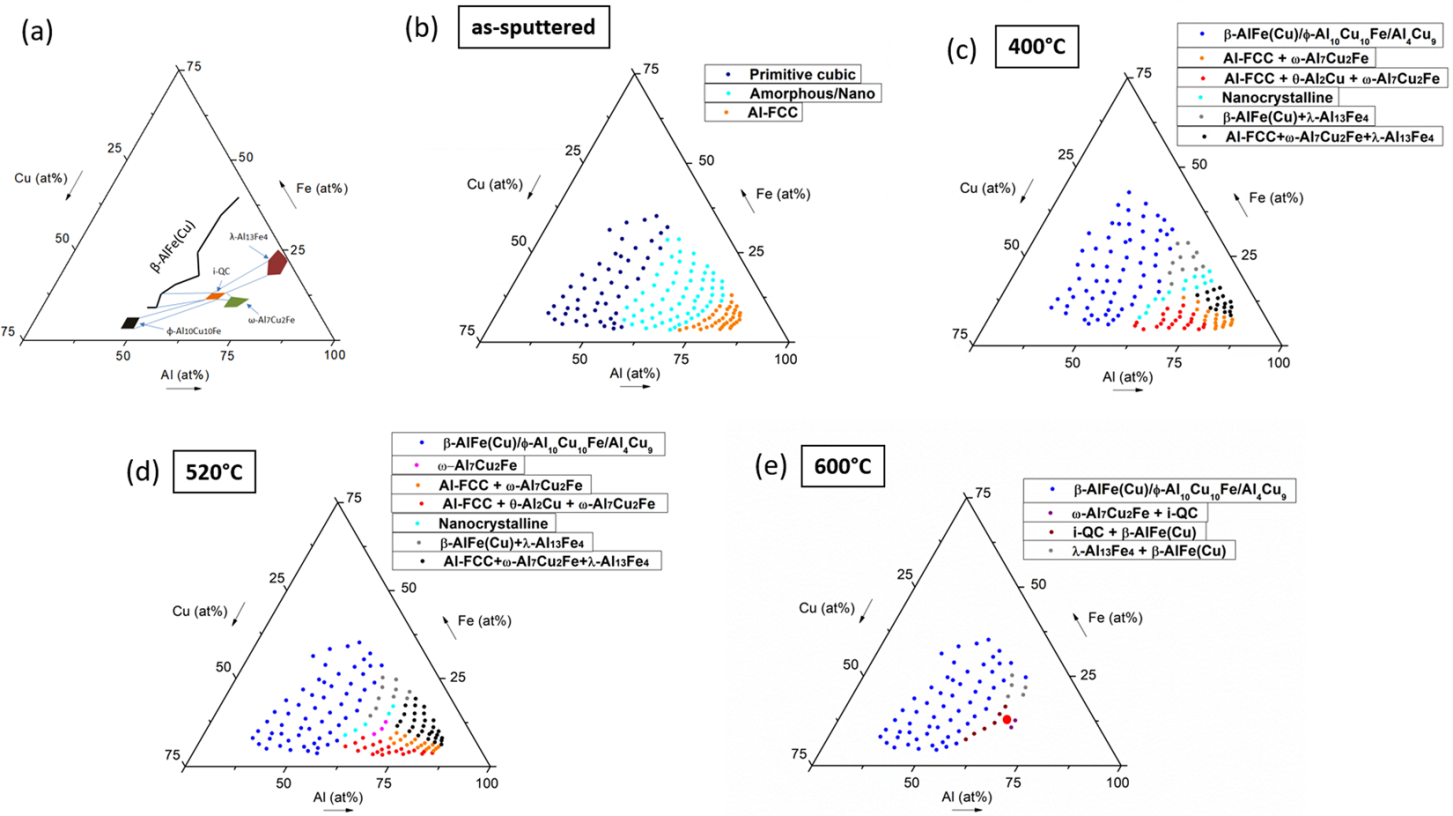
XRD mapping of the Al-Cu-Fe libraries. (**a**) Isothermal cut from the Al-Cu-Fe system at 700 °C, redrawn from reference^40^. In this figure, the quasicrystalline phase (i-QC) is shown along with the phases which form two-phase fields with it. Those phases are φ-Al_10_Cu_10_Fe, ω-Al_7_Cu_2_Fe, β-AlFe(Cu) and λ-Al_13_Fe_4_ and the two-phase fields are delineated by blue lines connecting the respective phase to the i-QC phase. (**b**) As-sputtered library: Primitive cubic, amorphous and Al-FCC phases. (**c**,**d**) Library annealed at 400 °C and 520 °C: β-AlFe(Cu), Al_4_Cu_9_, φ-Al_10_Cu_10_Fe, θ-Al_2_Cu, ω-Al_7_Cu_2_Fe, Al-FCC, Nanocrystalline, and λ-Al_13_Fe_4_ phases. (**e**) Library annealed at 600 °C: ω-Al_7_Cu_2_Fe, β-AlFe(Cu), φ-Al_10_Cu_10_Fe, Al_4_Cu_9_, i-QC and λ-Al_13_Fe_4_ phases. The Al-enriched alloys were severely oxidized at this temperature because they were close to their melting temperature. Phases in their diffractograms could not be properly identified and indexed.

Phase identification across the libraries was performed using X-ray diffraction (XRD) on the same alloy patches that were previously analyzed by EDX. This procedure allowed for phase identification in the Al-Cu-Fe libraries after being subjected to four different processing conditions, that is as-sputtered and annealed (Fig. 2b–e). A wide compositional spread was obtained for these libraries and a large portion of the Al-rich section of the Al-Cu-Fe ternary was fabricated and characterized in this work.

At 700 °C, the Al-Cu-Fe system is known to exhibit four phases forming two-phase regimes with the icosahedral QC^40^ (Fig. 2a). The two-phase fields are represented by the blue connecting lines (Fig. 2a). Figure 2a summarizes the current state of knowledge, to which we will now compare our findings: In the as-sputtered state, only three phases are identified (Fig. 2b). Al-FCC is the only phase indexed in the Al-rich compositions, while a primitive cubic phase is indexed in the Al-poorer compositions. The intermediate region shows XRD patterns with a low intensity signal and a broad diffraction band. While such diffractograms are typical of amorphous phases, the signal may include contributions from nanocrystals, which are difficult to distinguish from the amorphous phase from XRD alone. Sputtered Al-Cu-Fe films have been previously reported to be either amorphous^41,42^ or nanocrystalline^43^ in the as-sputtered state. The XRD patterns of each phase regime (shown in Fig. 2b–e) are presented in the supplementary XRD patterns S1 and all data are publicly available in our online data repository “MAP” (URL: http://materialsatlasproject.org/).

After annealing at 400 °C (Fig. 2c) most of the initially amorphous alloys have crystallized to phases such as ω-Al_7_Cu_2_Fe, θ-Al_2_Cu, and β-AlFe(Cu), which are expected to form in the Al-Cu-Fe system^3^. However, for the composition region between the QC and the λ-Al_13_Fe_4_ phase the diffractograms still show characteristics of an amorphous phase. At this temperature, no icosahedral quasicrystalline (i-QC) diffraction pattern could be observed in any of the alloys studied. The Al-poorer compositions (blue compositions in Fig. 2c) shows three dominant phases, namely: β-AlFe(Cu), Al_4_Cu_9_ and ϕ-Al_10_Cu_10_Fe. β-AlFe(Cu) is cubic primitive^3^ and the ϕ-Al_10_Cu_10_Fe has a diffraction pattern with the highest intense peak at the same position as β-AlFe(Cu) and Al_4_Cu_9_^44^. Due to the similar 2θ diffracting angles, it is difficult to distinguish the phases ϕ-Al_10_Cu_10_Fe and β-AlFe(Cu). However, as both are predicted to be present in the Al-Cu-Fe system as shown in reference^40^, they were both indexed in this work. Annealing at 520 °C (Fig. 2d) led to similar phase regimes as for 400 °C. The exception was the appearance of the single-phase region of the ω-Al_7_Cu_2_Fe phase and the shrinkage of the nanocrystalline field towards the known Al-Cu-Fe QC composition.

**Figure 3:**
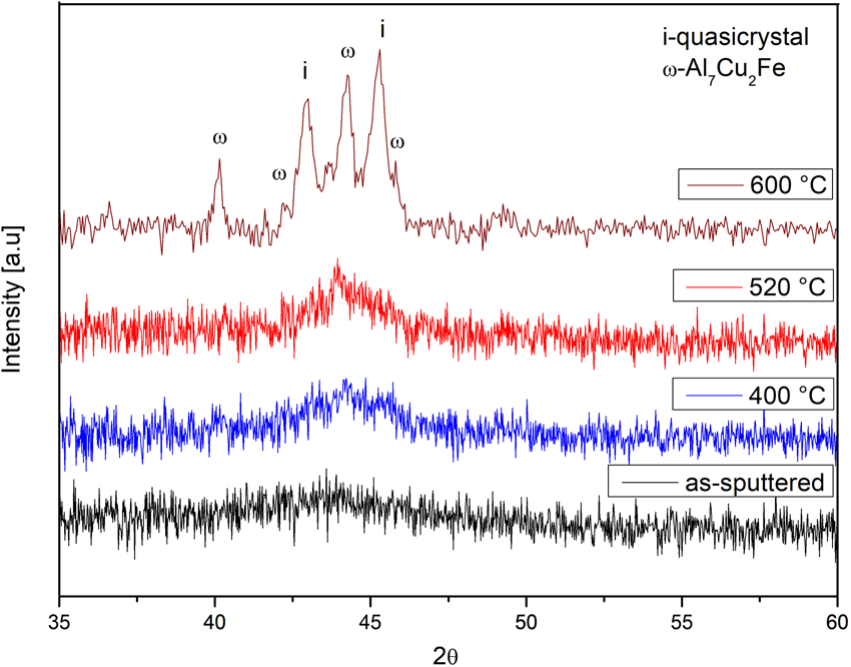
XRD patterns of compositions close to the predicted quasicrystalline phase, which showed an amorphous-like XRD pattern in the as-sputtered and annealed states. The as-sputtered film shows a shallow diffraction band. With increasing annealing temperature, the broad peak increases in intensity, indicating nano-crystallization during annealing. The corresponding diffractogram of the alloy annealed at 600 °C is also shown, confirming the presence of quasicrystalline phase along with ω-Al_7_Cu_2_Fe.

The highest annealing temperature considered was 600 °C, based on previous reports suggesting the QC formation around this temperature^29,41–43,45,46^. This library was annealed for two hours to provide sufficient time for solid-state transformations to occur. Most of the Al-rich compositions were severely oxidized, as they were exposed to a temperature close to or even higher (for the Al-Cu rich compositions) than their liquidus temperature. At these compositions, diffractograms could not be properly indexed. Such compositions were thus excluded from the phase mapping (Fig. 2e). Two phase regimes obtained in this work showed XRD patterns characteristic of the icosahedral QC phase. Both regions, however, consisted of a two-phase mixture (ω-Al_7_Cu_2_Fe + i-QC and β-AlFe(Cu) + i-QC). The QC phase is found between the ω-Al_7_Cu_2_Fe and β-AlFe(Cu) phases, forming two-phase mixture with both (Fig. 2a). The precise i-QC composition is located in between those phase regimes at a composition around Al_65_Cu_22_Fe_13_, in good agreement with the literature data^40^ reporting its chemical composition (Fig. 3e, at the position where a red circle is drawn). This icosahedral phase typically forms near the Al_62.5_Cu_25_Fe_12.5_ composition and was first discovered by A. P. Tsai^2^. It has drawn much attention due to its thermal stability, allowing it to be fabricated by conventional casting if a proper heat treatment is subsequently performed.

There is significant number of studies investigating the formation of QCs and intermetallic phases from sputtered and compositionally uniform Al-Cu-Fe films^29,41–43,45,47,48^. Most of these studies focus on the phase stability of the samples in the as-sputtered state and after annealing at different temperatures. In this work, we expand the amount of data related to the composition range and processing conditions to correlate phase stability with composition and temperature.

In previous studies, the atomic structure of the as-sputtered Al-Cu-Fe based films depends on the thickness of the film and on the temperature of the substrate^41–43^. Amorphous films were obtained when the temperature of the substrate was cooled down to liquid nitrogen temperature^41^ or if the film was sufficiently thin that there was no re-heating of the growing film due to atomic bombardment resulting from the sputtering process^43^. What is common to all these studies is that the XRD diffractograms of the as-sputtered films would always feature a broad low intensity diffraction band, from which amorphous and nanocrystalline structure cannot be distinguished. In such cases TEM analysis would be necessary. In the present work we reveal that the formation of an amorphous/nanocrystalline microstructure in as-sputtered Al-Cu-Fe films is not restricted to the composition region of the i-QC phase but it can cover a larger composition range from around 55 at.% to 77%at. of Al content.

Table 1 summarizes literature findings on the phase stability of Al-Cu-Fe films at different temperatures. Several complex phase transformations take place between 200 °C and 850 °C and different results were reported in the above-mentioned studies. In this table, “I” represents intermetallics, “QC” stands for quasicrystal, “P.” is primitive, “NanoCrys” stands for nanocrystalline grain structure and “R” is a rhombohedral phase, approximant of the quasicrystal. Table 1 shows that even for identical nominal compositions fabricated by magnetron sputtering, different phases were reported for very similar annealing temperatures^41,42^. Also, when very close nominal compositions were fabricated, different phases were again observed for identical annealing temperatures^43,46^. These differences are a direct consequence of the narrow QC phase field and due to the number of intermetallic phases which surround this phase. From inspecting the 600 °C or 700 °C isothermal sections of the ternary Al-Cu-Fe^40,49^, it becomes evident that even slight changes in the composition can completely change the microstructure of the alloy (Fig. 2a). Controlling the sputtering deposition parameters to obtain single composition films with the exact desired atomic composition is not a straightforward task because even in a well-controlled sputtering environment small variations of the chemical composition of the films are inherent to the process. The method of combinatorial sputtering avoids this issue, as hundreds of alloys are fabricated at the same time, increasing the probability of obtaining the desired composition at some point of the library.

**Table 1:** Summary of the reported phase stability in Al-Cu-Fe sputtered films and annealed at various temperatures.

Ref.	%at.	Substrate Cooling	Thickness	As-sputtered	400 °C	450 °C	550 °C	600 °C	750 °C	850 °C
Chien *et al*.^41^	Al65Cu20Fe15	N2 liquid	10 µm	Amorphous	—	Cubic P.	—	QC	—	—
Ding *et al*.^42^	Al65Cu20Fe15	—	10 µm	Amorphous	QC + I.	—	QC + I	—	QC	—
Widjaja *et al*.^43^	Al64Cu23Fe13	—	150–200 nm	NanoCrys	Cubic P. + I	—	—	—	—	—
Daniels *et al*.^29^	Al60Cu22Fe10O8	—	10 µm	Amorphous/NanoCrys	—	Approximant R	—	—	—	QC
Vollnhofer *et al*.^45^	Al61Cu25Fe10B4	—	5 µm	Amorphous/NanoCrys	—	—	—	QC + Cubic P.	—	—
Bonasso *et al*.^46^	Al62Cu25.5Fe12.5	—	100 nm	Amorphous/NanoCrys	Amorphous/NanoCrys	NanoCrys + Cubic P. +α-Al	—	QC + Cubic P.	—	—

The findings presented here show that the combinatorial method is appropriate for identifying QC alloys and that it could be applied for discovering new QC-forming systems in a fast and efficient fashion. The method also provides valuable information concerning the annealing temperatures necessary to reveal the formation of the intermetallic phases in Al-Cu-Fe sputtered films. We show that annealing at 400 °C is sufficient to reveal all the intermetallic phases (in the Al-rich region of this system), with exception of the λ-Al_13_Fe_4_ and the QC, which requires higher annealing temperatures (520 °C and 600 °C, respectively).

### Nanocrystalline microstructure close to the quasicrystal composition

As shown in the libraries phase regimes identified in this study (Fig. 2b–e), there were compositions that showed an amorphous/nanocrystalline-like diffraction pattern (Fig. 3). Specifically, after annealing at 400 °C and 520 °C those compositions are found close to the QC and λ-Al_13_Fe_4_ phases. Only after annealing at 600 °C those compositions would now have defined peaks in the diffraction patterns (Fig. 3). We assume that in the as-sputtered state, the amorphous phase is dominant in those compositions. In the 400 °C/520 °C annealed samples, nano-crystallization and subsequently at 600 °C, crystal growth takes place, as concluded from the increase in intensity of the broad diffraction bands from as-sputtered to 400 °C/520 °C, and finally the appearance of sharp peaks at 600 °C (Fig. 3).

**Figure 4:**
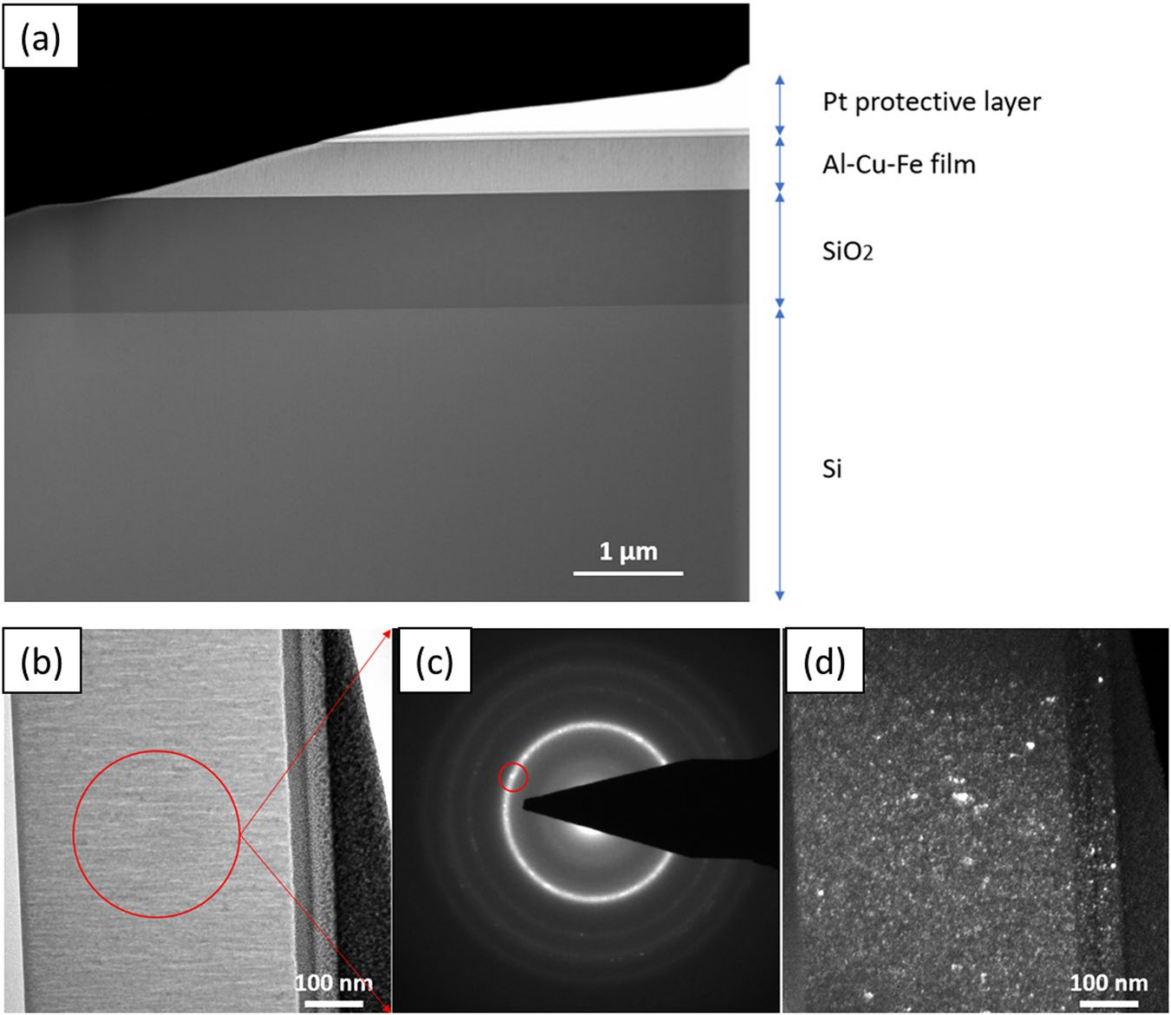
(**a**) STEM micrograph showing the layered structure of the sample corresponding to the composition outlined in Fig. 2a, prepared by FIB. The micrograph shows the Si substrate and the SiO_2_ diffusion barrier. The Al-Cu-Fe alloy shown here was taken from the library annealed at 400 °C. A Pt layer was applied on the Al-Cu-Fe film surface for protection during sample preparation. (**b**) TEM bright field micrograph of the Al-Cu-Fe film showing the area where the electron diffraction pattern was measured. The film displays columnar growth. (**c**) Selected area electron diffraction (SAED) pattern of the Al-Cu-Fe film. A very fine microstructure is observed from the pattern due to reflections such as the one highlighted by the red circle. (**d**) TEM dark field micrograph from the reflection highlighted in (**c**), confirming the presence of nanocrystallinity.

To confirm the presence of nanocrystals in the annealed alloys, a patch annealed at 400 °C (from the same composition as the diffractogram in Fig. 3) was analyzed by TEM after FIB preparation (Fig. 4). Three layers can be observed on the Si wafer, namely: the oxide layer diffusion barrier (SiO_2_) with an average thickness of 1 µm, the Al-Cu-Fe film of about 500 nm and a Pt layer that was added for protection of the film during sample preparation (Fig. 4a).

TEM analysis comprised a bright field micrograph of the Al-Cu-Fe film with the respective selected area where electron diffraction analysis was made (Fig. 4b). The diffraction pattern (Fig. 4c) shows a ring profile with several diffracting planes suggesting presence of nanocrystalline grain structure. The presence of a fraction of amorphous structure is not ruled out. The red circle outlined on the diffraction pattern shows the diffracting planes selected for the dark field micrograph (Fig. 4d) which confirms that the sample consists of a very fine grain structure (with grain sizes ranging from 2 to 10 nm).

**Figure 5:**
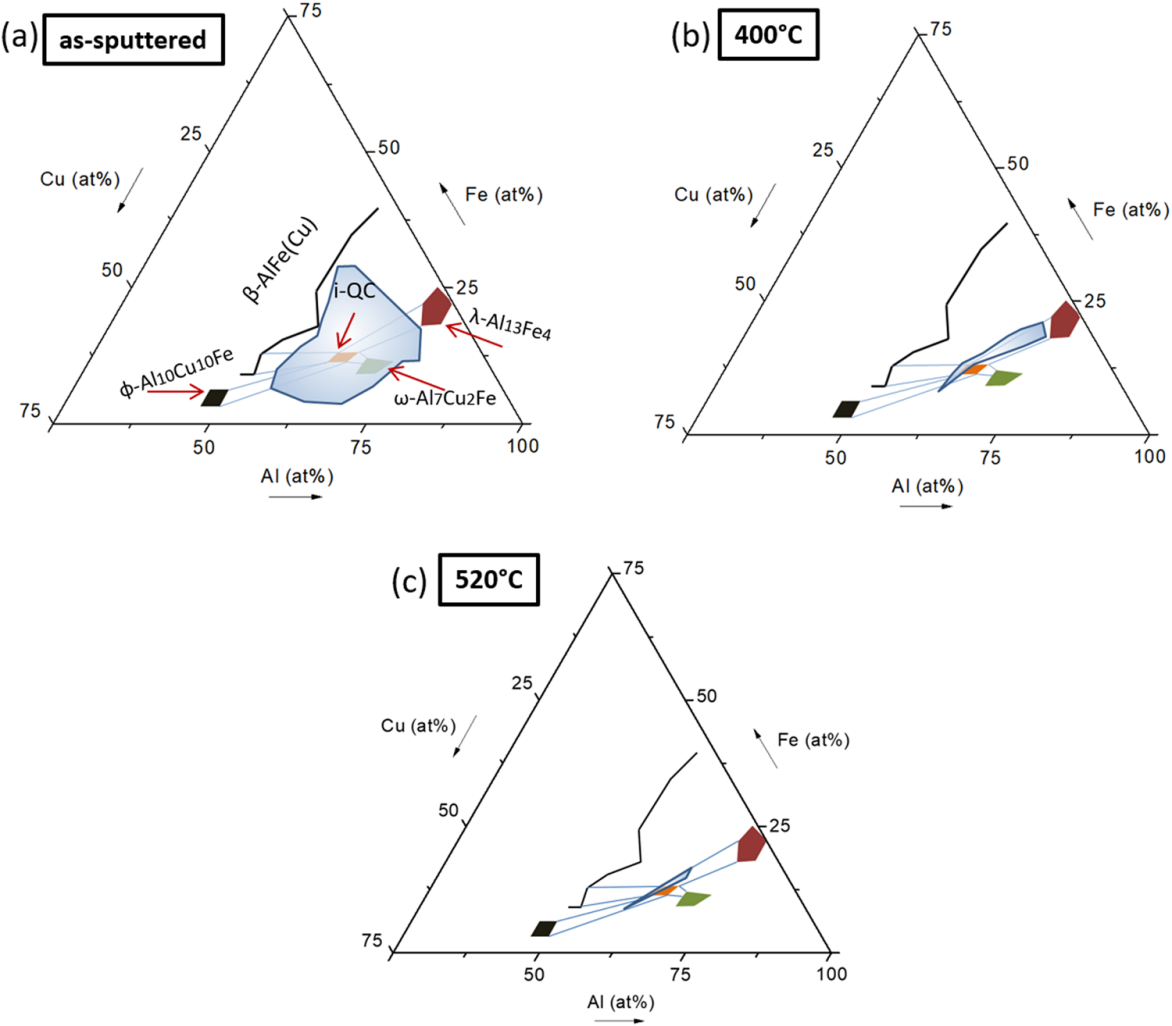
Formation of amorphous and nanocrystalline phases on the as-sputtered and annealed libraries. (**a**) Isothermal cut from the Al-Cu-Fe system at 700 °C, redrawn from reference^40^ (Republished with permission of Royal Society of Chemistry; permission conveyed through Copyright Clearance Center, Inc.). In this figure, the quasicrystalline phase (i-QC) is shown along with the phases that form two-phase fields with it. Those phases are φ-Al_10_Cu_10_Fe, ω-Al_7_Cu_2_Fe, β-AlFe(Cu) and λ-Al_13_Fe_4_. The two-phase fields are delineated by blue lines connecting them to i-QC. Compositions which exhibited amorphous XRD patterns in the as-sputtered libraries are represented as a blue-shaded area. (**b**,**c**) Compositions with nanocrystalline grain structure after annealing at 400 °C and 520 °C, respectively. These are the compositions between i-QC and λ-Al_13_Fe_4_, of which both are phases with a high degree of polytetrahedral atomic structure.

Plotting the compositions studied in this work that showed amorphous/nanocrystalline-like XRD patterns on the Al-Cu-Fe isothermal cut, from reference^40^ (Fig. 5a–c) we would notice an interesting trend. Through annealing at 400 °C, the wide compositional range where the amorphous/nanocrystalline phase is found shrinks into the i-QC/λ-Al_13_Fe_4_ two-phase region. Increasing the annealing temperature to 520 °C further reduces this range to compositions close to the QC composition.

This behavior can be explained in terms of nucleation and growth of phases with a high degree of polytetrahedral ordering elements in the atomic structure^36,50–52^. Directly^53–57^ and indirectly^36,50^ it has been shown that the liquid comprises icosahedral short range order^58^. This atomic configuration of metallic melts is thought to create an additional barrier for nucleation of high-symmetry crystalline phases, such as cubic phases, because the icosahedral order has to be broken to rearrange into the lattice positions of the crystalline structure. However, in the case of QCs and approximants such as λ-Al_13_Fe_4_, the icosahedral atomic configurations are similar to the ones in the melt, leading to a lower barrier for nucleation and reflecting in a lower interfacial energy between the liquid (amorphous phase in the case of the libraries studied here) and the solid phases.

Holland-Moritz *et al*.^50^ have studied the nucleation of icosahedral and decagonal QCs and intermetallic phases (λ-Al_13_Fe_4_, µ-Al_5_Fe_2_ and β-CsCl) in Al-Cu-Fe and Al-Cu-Co alloys and the phases that had the lowest values of interfacial energy between the solid nucleus and the liquid phase were the i-QC and the λ-Al_13_Fe_4_ (in ascending order). This lead to a finer grain structure for these phases, since more supercritical nuclei can form at a higher rate from the amorphous phase, which is what we experimentally observe here, confirming results obtained in previous work^36,50,51,59^. In addition, it was shown that growth rates of QCs and phases with polytetrahedral atomic configuration are smaller by more than one order of magnitude than the ones observed in pure metals and solid solutions^52^. This is also in accordance with the results obtained here: Due to sluggish growth of the i-QC phase, a nanocrystalline grain structure is retained even after annealing at 520 °C (Fig. 5c).

### Preparation of Al-Cu-Fe-Cr libraries

Al-Cu-Fe-Cr libraries were fabricated using a similar setup as for the ternary samples. The main difference lies in the target configuration. Three outer targets (Cr, Fe and Cu) are positioned in a tetrahedral configuration at an angle of 30 degrees towards the vertical (in the same way as shown in Fig. 1). The fourth target (Al) is in the center. Depositing Al from the center sputtering gun results in an approximately uniform distribution of the Al concentration across the library. Two libraries were fabricated in this study with a nominal Al content of 65 at.% and 70 at.%, which will be referred to as Al65CuFeCr and Al70CuFeCr, respectively. The three outer targets produced a compositional gradient of Cr, Fe and Cu across the library, yielding a number of approximately 150 alloys per library. The as-sputtered libraries were characterized using EDX and subsequently annealed at 600 °C for 2 h. The annealing parameters were chosen in accordance with the previous results obtained for the ternary libraries, which exhibited the QC phase only at this temperature.

### QC phase identification in Al-Cu-Fe-Cr libraries

**Figure 6:**
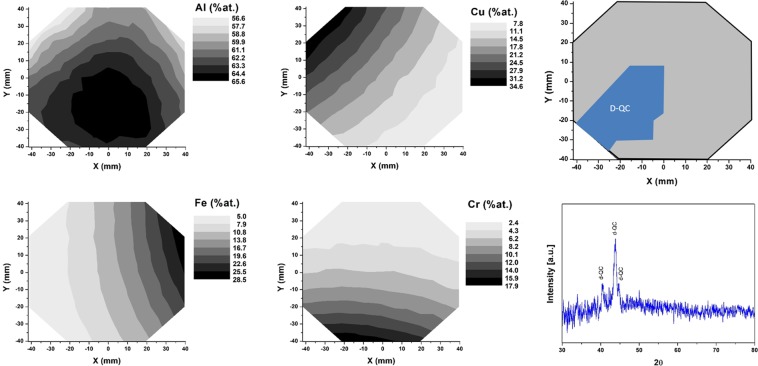
Library Al65CuFeCr: Compositional distribution of Al, Cu, Fe and Cr across the library. The decagonal quasicrystalline phase field at 600 °C is indicated in blue. An example diffractogram used to identify the decagonal phase is provided.

**Figure 7:**
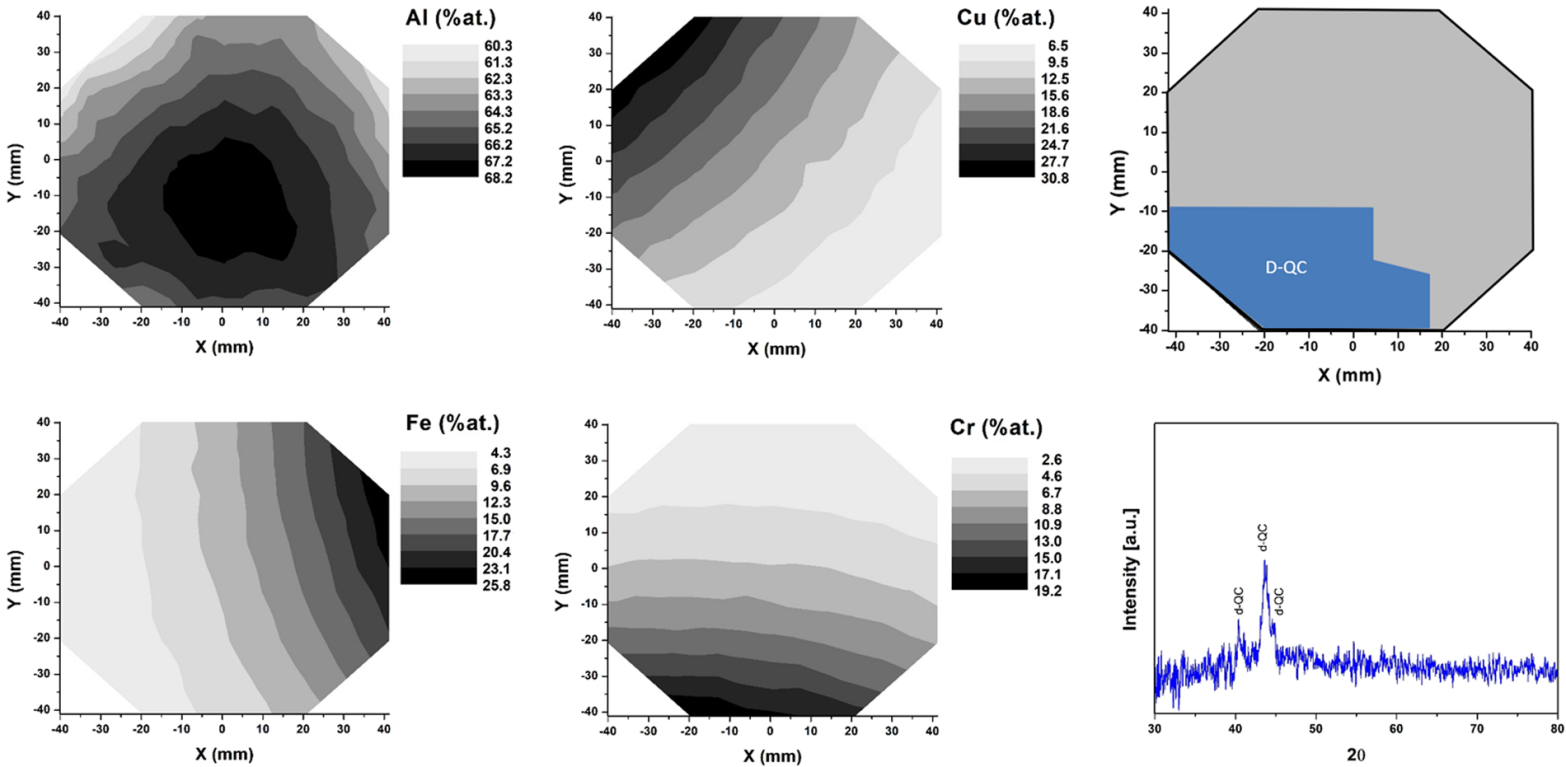
Library Al70CuFeCr: compositional gradient of Al, Cu, Fe and Cr within the wafer, decagonal quasicrystalline phase field (at 600 °C) and an example of XRD pattern used to identify the decagonal phase.

The compositional distributions of Al, Cu, Fe and Cr obtained in the two libraries (cf. Figures 6 and 7) show that both libraries exhibit an approximately constant Al content at the center, which is slightly lower than the nominal content.

After annealing, XRD analysis reveals a wide decagonal QC composition range for both libraries (Figs 6 and 7, outlined in blue in the wafer drawings). Corresponding example diffractograms are shown (Figs 6 and 7), displaying the three characteristic peaks of the decagonal phase^35^. It is important to point out that the identified QC phase field can be attributed to both the decagonal phase and the orthorhombic approximant of this phase^35^. By means of XRD only it is not possible to distinguish between them. However, several publications report that when fabricating an Al-Cu-Fe-Cr QC alloy, a decagonal phase can form if high cooling rates are applied and/or if proper annealing is conducted^5,28,33,35,60^. Dong *et al*.^35^ could demonstrate the formation of the approximant crystalline phase only after annealing at 927 °C. Annealing sputtered films at lower temperatures (450 to 750 °C) lead to the formation of QC phases^28,33,60^. Accordingly, the phase field outlined (Figs 6 and 7) should be constituted of the decagonal phase, rather than the orthorhombic approximant. In this work, the library compositions that showed other crystalline phases as dominant will not be discussed.

The results obtained here show for the first time the composition range across which the decagonal QC phase can be obtained in the Al-Cu-Fe-Cr system for an Al content ranging from 65 to 70 at.%. The Al65CuFeCr library revealed that the QC phase can be found in the range Al_63–65_Cu_12–22_Fe_5–11_Cr_6–15_. The Al70CuFeCr library revealed the QC phase field as Al_65–67_Cu_8–20_Fe_4–11_Cr_8–16_. Both these ranges were found for samples annealed at 600 °C.

This finding suggests that the Al-Cu-Fe-Cr decagonal QC has an abnormally large compositional range of formation, unlike most known QC-forming systems^13^, which exhibit rather narrow composition ranges of formation. This is very interesting for a practical application of these materials. Large composition ranges provide more flexibility during complex fabrication processes such as gas-atomization, physical vapor deposition and thermal spraying, because the control of chemical composition will not be as critical as it usually is for producing QCs.

**Figure 8:**
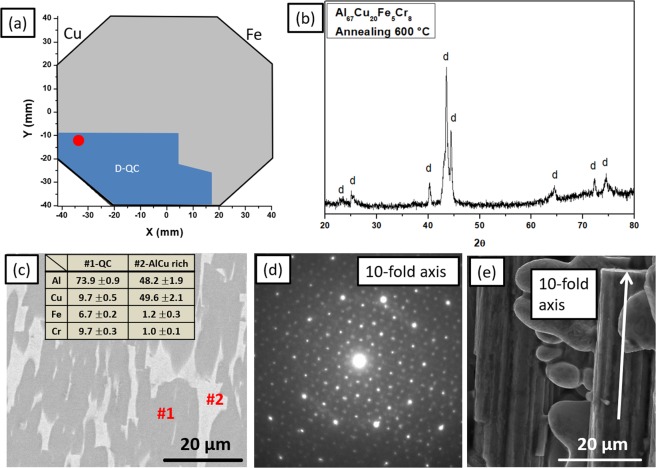
(**a**) Al_67_Cu_20_Fe_5_Cr_8_ composition on the Al70CuFeCr library. (**b**) Diffractogram of the arc-melted Al_67_Cu_20_Fe_5_Cr_8_ alloy after annealing at 600 °C. (**c**) SEM-BSE micrograph of the annealed sample showing the presence of two phases: decagonal quasicrystal (phase #1) and an Al-Cu-rich phase (phase #2). (**d**) SAED pattern of the quasicrystalline phase, displaying ten-fold rotational symmetry. (**e**) Columnar morphology of a single decagonal quasicrystal. This phase typically grows in such a columnar fashion along the ten-fold axis of symmetry.

In previous work, a QC phase in an Al_67_Cu_20_Fe_5_Cr_8_ (at.%) melt-spun alloy was reported as the dominant phase in the microstructure^5^. In the present work one of the Al70CuFeCr library´s patches, with the same composition (Al_67_Cu_20_Fe_5_Cr_8_), was found to be within the decagonal phase field (outlined in red, Fig. 8a). To validate the combinatorial method used here to find new quasicrystalline compositions, this alloy was fabricated by arc-melting and annealed at 600 °C. The diffractogram of the annealed alloy (Fig. 8b), shows that the dominant phase of this sample is either QC or an approximant. The SEM-BSE micrograph shows that the sample consists of two phases (Fig. 8c). The dominant phase is the decagonal QC, as confirmed by TEM studies using selected area electron diffraction pattern (SAED) (Fig. 8d), which reveals the ten-fold rotational symmetry. The secondary phase is an Al-Cu rich phase, which was also found in a small fraction in the previously reported melt-spun alloy^5^. The formation of this phase can be explained from Fig. 8a, which shows where the Al_67_Cu_20_Fe_5_Cr_8_ composition is located on the wafer. This alloy is close to the boundary between the QC forming field and other crystalline phases. Several different Al-Cu based phases can be found in the vicinity of this composition^61^ and the Al-Cu-rich phase has not been further analyzed here. An SEM-SE image of a fracture site of the alloy (Fig. 8e) is also shown, in which the columnar growth of the decagonal phase towards the ten-fold rotational symmetry element of this atomic structure can be identified.

## Conclusions

Combinatorial strategies were used to fabricate and characterize ~140 Al-Cu-Fe and ~300 Al-Cu-Fe-Cr distinct compositions. The alloys were analyzed in the as-sputtered state and after annealing at 400 °C, 520 °C and 600 °C. Such large number of consistent data allows us to evaluate the stability range of the QC phase and nearby approximant phases. The results show that the combinatorial strategy applied here is appropriate for identifying and possibly discovering QC-forming alloys and systems. The compositional range across which the decagonal Al-Cu-Fe-Cr forms was found for an Al content up to 70 at.%. Furthermore, conclusions about the crystallization mechanism of Al-Cu-Fe based phases can be drawn as larger fractions of the considered composition ranges are amorphous in the as-sputtered state. Specifically, phases with polyhedral atomic configuration such as the QC and λ-Al_13_Fe_4_ phases exhibit higher nucleation rates as compared to the other phases in the system such as ω-Al_7_Cu_2_Fe, θ-Al_2_Cu, and β-AlFe(Cu).

## Methods

Al-Cu-Fe and Al-Cu-Fe-Cr compositional libraries were fabricated by confocal DC magnetron co-sputtering (AJA International ATC 2200) using elemental targets with purities above 99.95%. For the ternary libraries the targets were placed in a tetrahedral configuration at an angle of 30 degrees from the vertical (Fig. 1a). A similar configuration was used for the quaternary samples, but the fourth target, Al, was placed at the central position, parallel to the wafer. The substrates used were 100 mm diameter Si wafers with a 1 µm SiO_2_ thermally grown oxide on the surface. This oxide layer was used to prevent inter-diffusion between Si and the films on subsequent thermal treatments. The substrate to target distance was 67 mm and a physical mask was placed on the Si-wafer surface to obtain separated alloy patches of 1.8 mm diameter each. Prior to the co-sputtering process, a base pressure of <10^−5^ Pa was attained, which was then increased for the sputtering process to 0.3 Pa of ultra-high purity Ar. The compositional spread was controlled by adjusting the power applied to the targets. For the ternary libraries, the target central composition of Al_62.5_Cu_25_Fe_12.5_ was aimed for to tune the powers applied to the targets, which were 120 W, 12 W and 8 W for Al, Fe and Cu respectively. The deposition process was carried out for 2h50min to obtain 500 nm-thick films. For the quaternary libraries, the target central compositions were Al_65_Cu_20_Fe_10_Cr_5_ and Al_70_Cu_17_Fe_8_Cr_5_. The deposition processes were carried out for about 55 minutes and resulted in 500 nm-thick films.

The as-sputtered libraries were characterized using an automated energy dispersive X-ray spectroscopy (EDX) detector from Oxford Instruments, attached to a Zeiss Sigma VP Field Emission scanning electron microscope. Not all patches were analyzed.

After EDX analysis, the samples were annealed for different times and at different temperatures: 400 °C/1 h, 520 °C/1 h and 600 °C/2 h for the ternary libraries (Fig. 1d) and 600 °C/2h for the quaternary libraries. A base pressure of 7.7 Pa was achieved and the thermal treatment was performed under ultra-high purity Ar flow.

X-ray diffraction (XRD) analysis was performed using scanning XRD (Rigaku Smartlab) with Cu Kα radiation and a 2 mm beam mask; the same patches analyzed by EDX were analyzed by XRD. One alloy patch of the Al-Cu-Fe library was analyzed by transmission electron microscopy (TEM) with a FEI Tecnai Osiris, operating at 200 kV. The sample was prepared by Focused Ion Beam (FIB) using a FEI Helios NanoLab 660 SEM/FIB.

The Al_67_Cu_20_Fe_5_Cr_8_ alloy bulk sample was produced by arc-melting high purity (>99.95%) elements under argon atmosphere and then annealed at 600 °C, for 10 h under argon atmosphere. After thermal treatment the bulk sample was characterized by XRD using a Bruker D8 ADVANCE with Cu-Kα radiation. Scanning and transmission electron microscopy, SEM and TEM, were used for microstructural characterization, using a Philips XL-30 FEG SEM and a FEI TECNAI G2 F20 TEM operating at 200 kV. SEM sample was prepared by grinding up to a 1500 grit sand paper and then by polishing using a 1 μm alumina. TEM sample was obtained by crushing the material into a fine powdery form by use of a ceramic mortar. A suspension of the powdery sample and methyl alcohol was then prepared and dripped on a carbon grid, which was ready for TEM analysis after evaporation of the methyl alcohol.

## Supplementary information

Supplementary XRD patterns – S1

## Data Availability

The datasets generated in this work are available from the corresponding author on reasonable request. The XRD and EDX data from the combinatorial experiments are publicly available at http://materialsatlasproject.org/.
