# Supplementary material for: Formation and stability of complex metallic phases including quasicrystals explored through combinatorial methods
Source: Sci Rep. 2019 May 9;9:7136. doi: 10.1038/s41598-019-43666-w (PMC6509252; doi:10.1038/s41598-019-43666-w)
Supplement: Supplementary file 1 — Supplementary XRD patterns – S1 [file 41598_2019_43666_MOESM1_ESM.docx]

**Supplementary XRD patterns – S1**

**Formation and stability of complex metallic phases including quasicrystals explored through combinatorial methods**

Witor Wolf^*,a,b^, Sebastian A. Kube^c^, Sungwoo Sohn^c^, Yujun Xie^c^, Judy J. Cha^c^, B. Ellen Scanley^d^, Claudio S. Kiminami^e^, Claudemiro Bolfarini^e^, Walter J. Botta^e^ and Jan Schroers^c^

^a^Programa de Pós-Graduação em Ciência e Engenharia de Materiais, Universidade Federal de São Carlos, Rod. Washington Luiz, Km 235, São Carlos, SP, 13565-905, Brasil.

^b^Departamento de Engenharia Metalúrgica e de Materiais, Universidade Federal de Minas Gerais, Av. Antônio Carlos, 6627, Belo Horizonte, MG, 31270-901, Brasil.

^c^Department of Mechanical Engineering and Materials Science, Yale University, New Haven, Connecticut, 06511, USA.

^d^Department of Physics, Southern Connecticut State University, New Haven, Connecticut, 06515, USA.

^e^Departamento de Engenharia de Materiais, Universidade Federal de São Carlos, Rod.Washington Luiz, Km 235, São Carlos, SP, 13565-905, Brasil.

**XRD patterns – as-sputtered condition. (Amorphous/Nano XRD on the manuscript).**





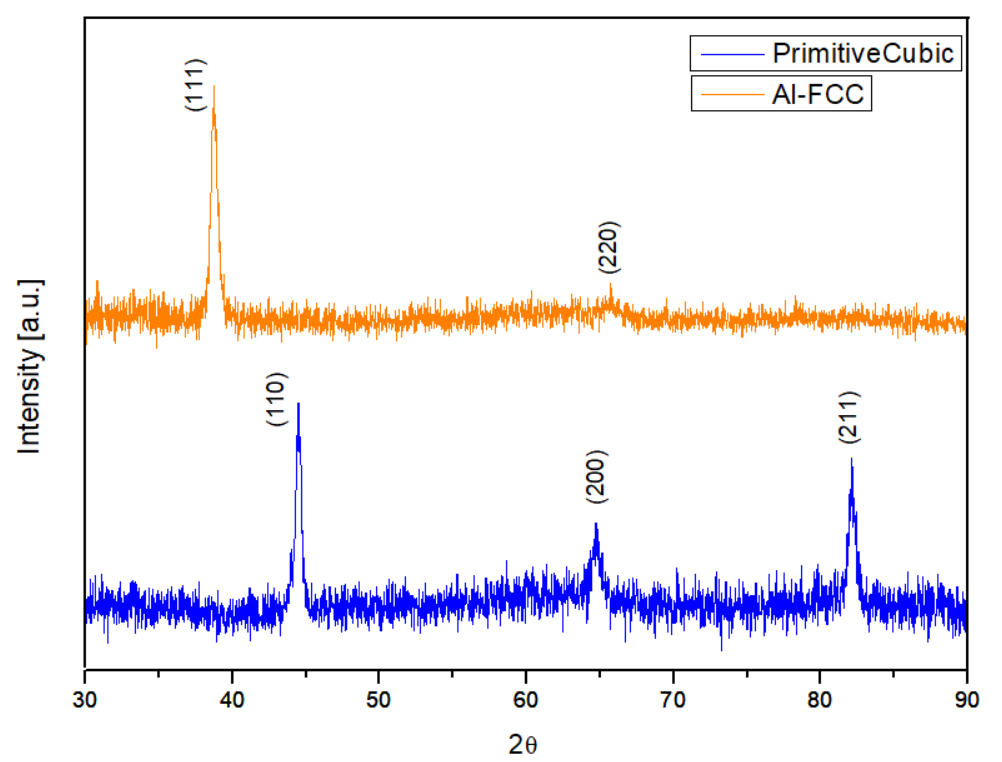


Figure 1 Phase regimes: blue and orange. Light-blue was already plotted on the manuscript.

**XRD patterns – annealing at 400 °C. (Nanocrystalline XRD on the manuscript).**





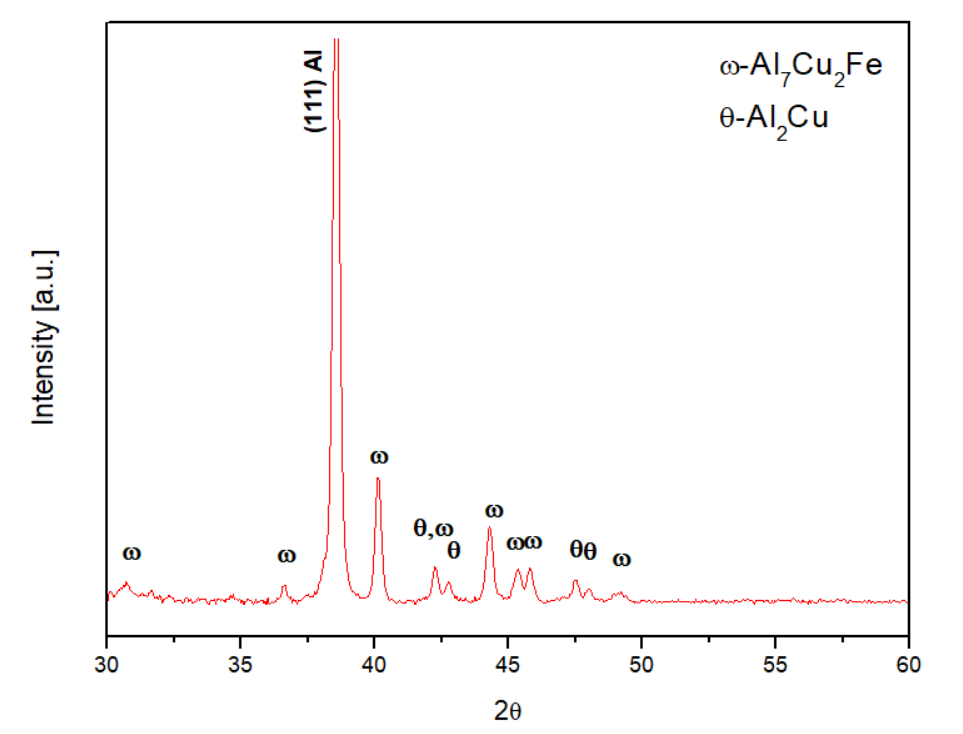


Figure 2 Phase regime: red.


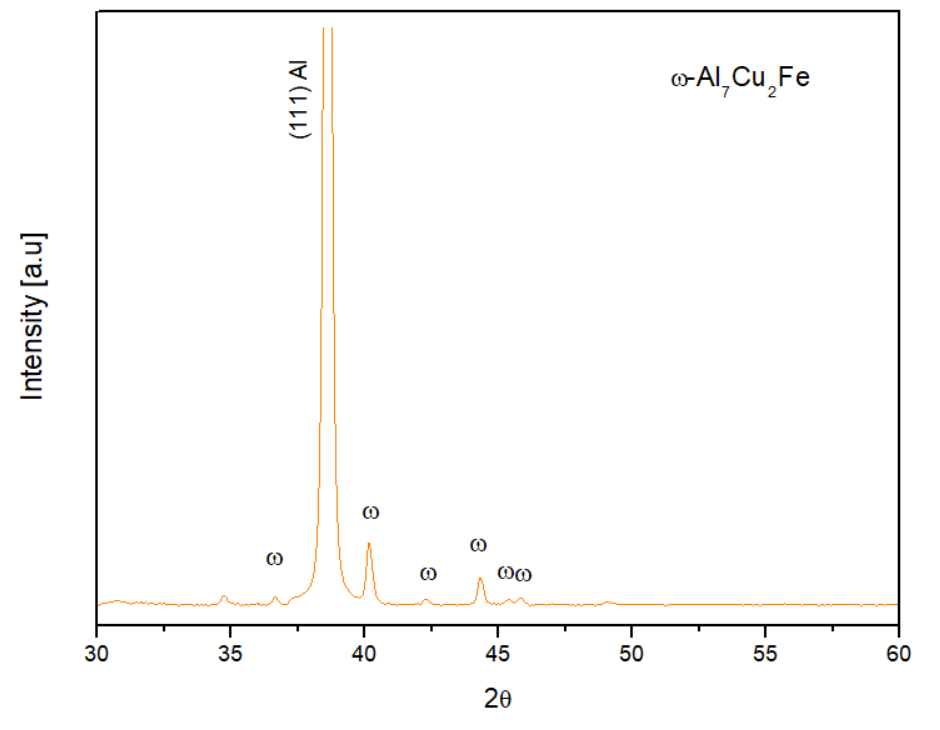


Figure 3 Phase regime: orange.


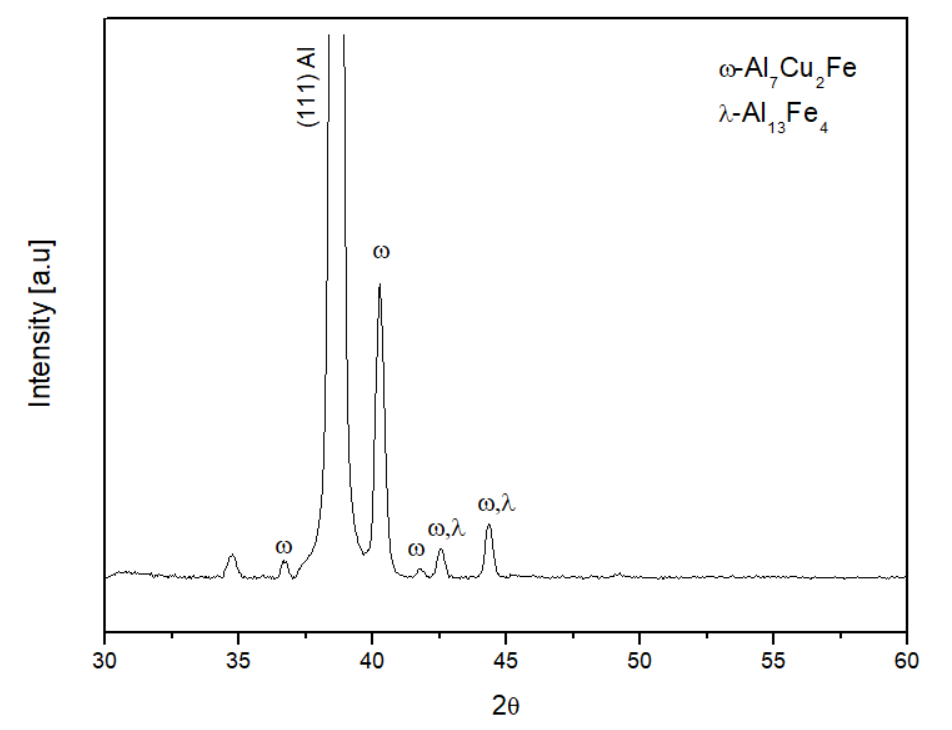


Figure 4 Phase regime: black.


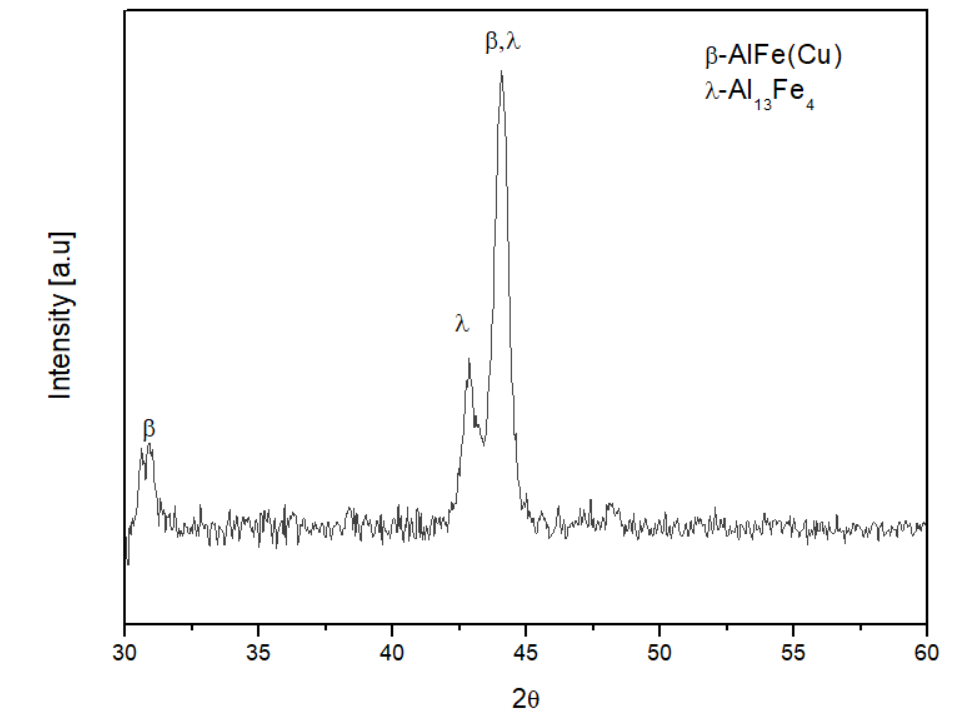


Figure 5 Phase regime: gray.


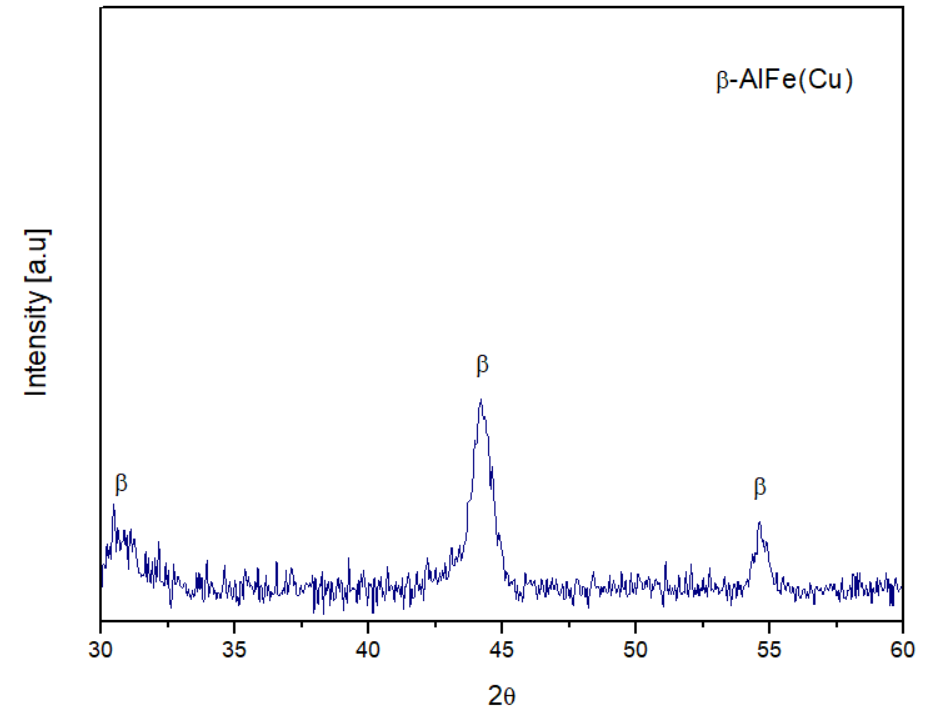


Figure 6 Phase regime: blue.


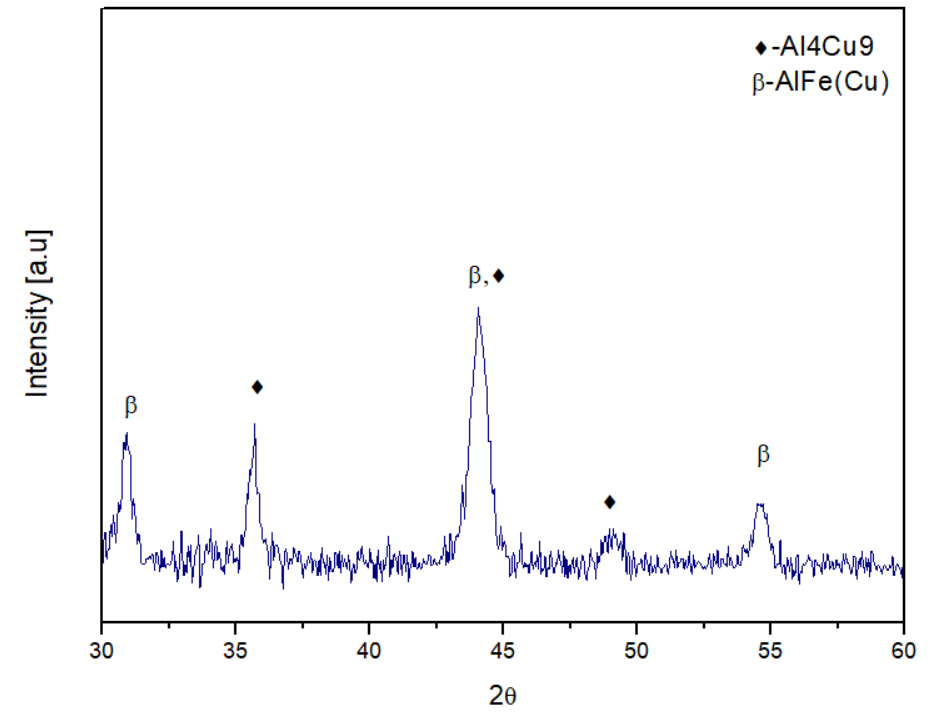


Figure 7 Phase regime: blue.


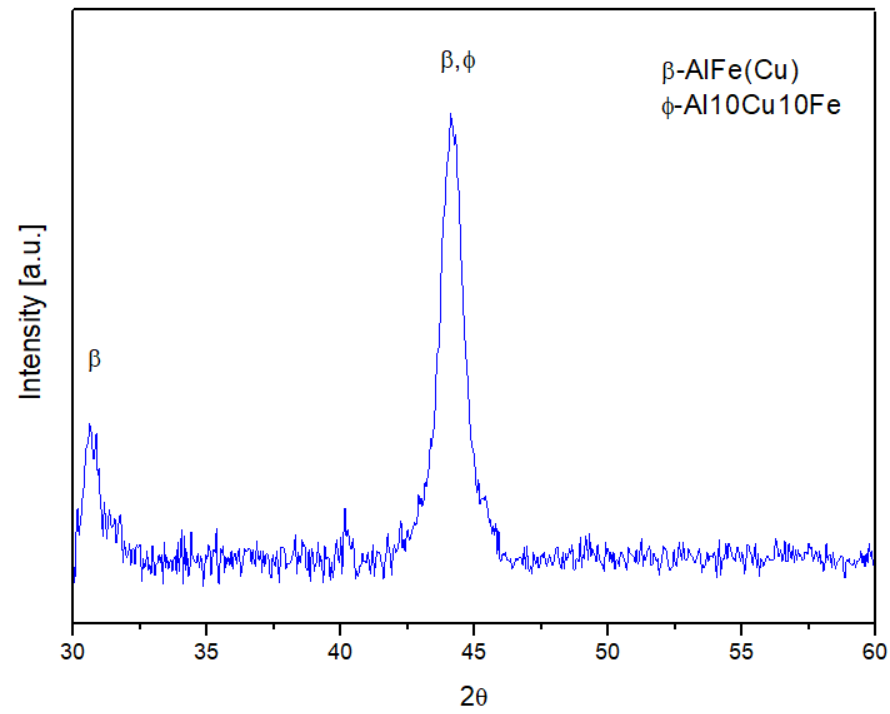


Figure 8 Phase regime: blue.

**XRD patterns – annealing at 520 °C. (Nanocrystalline XRD on the manuscript).**


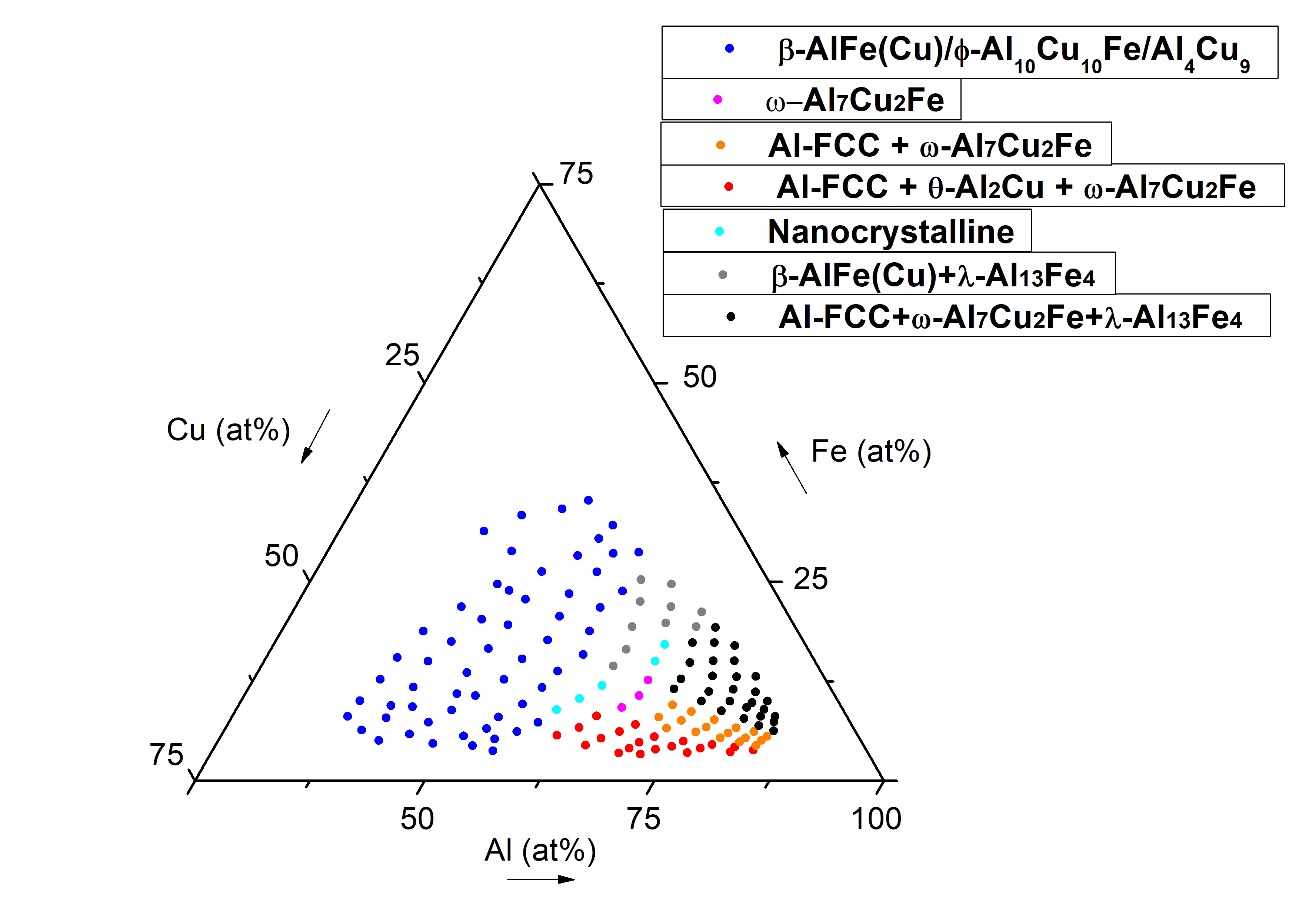


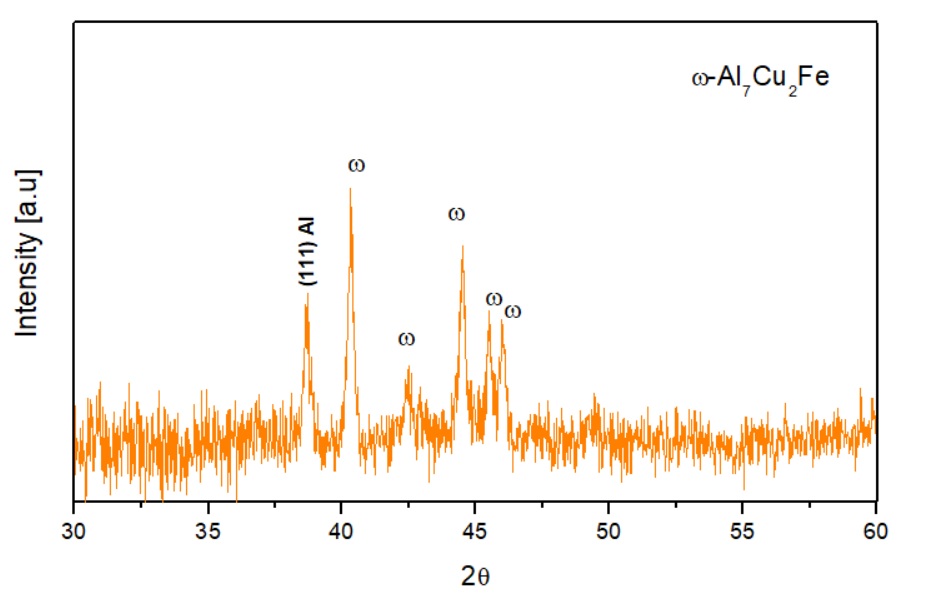


Figure 9 Phase regime: orange.


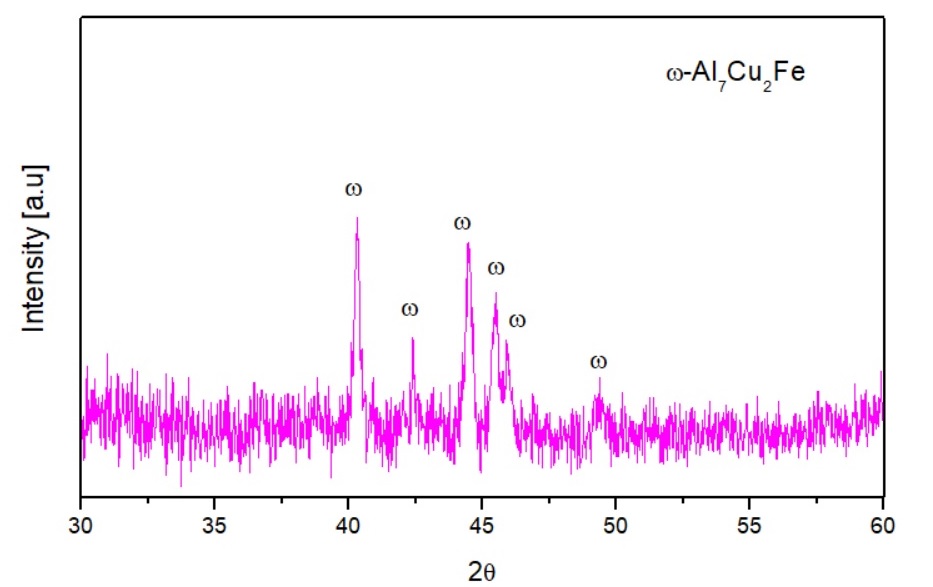


Figure 10 Phase regime: violet.


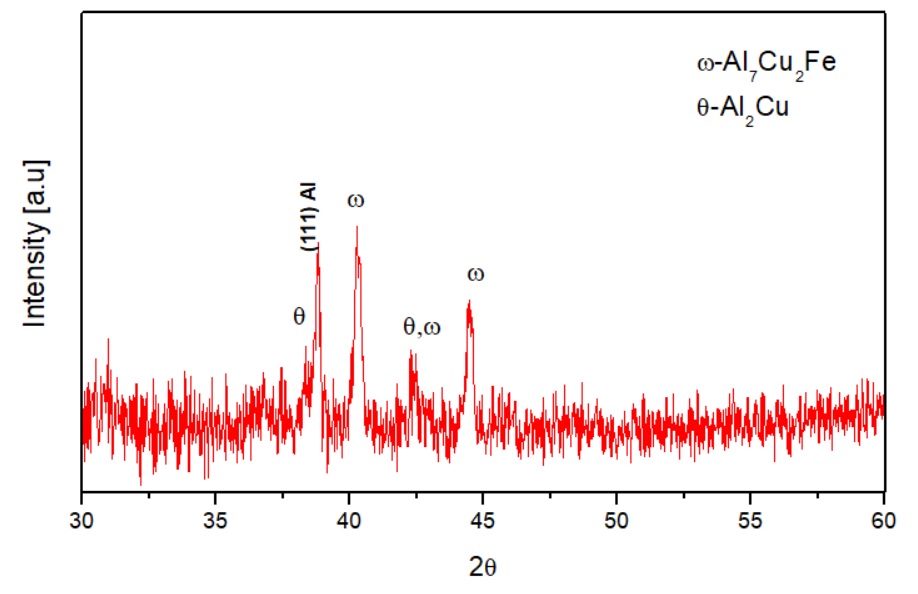


Figure 11 Phase regime: red.


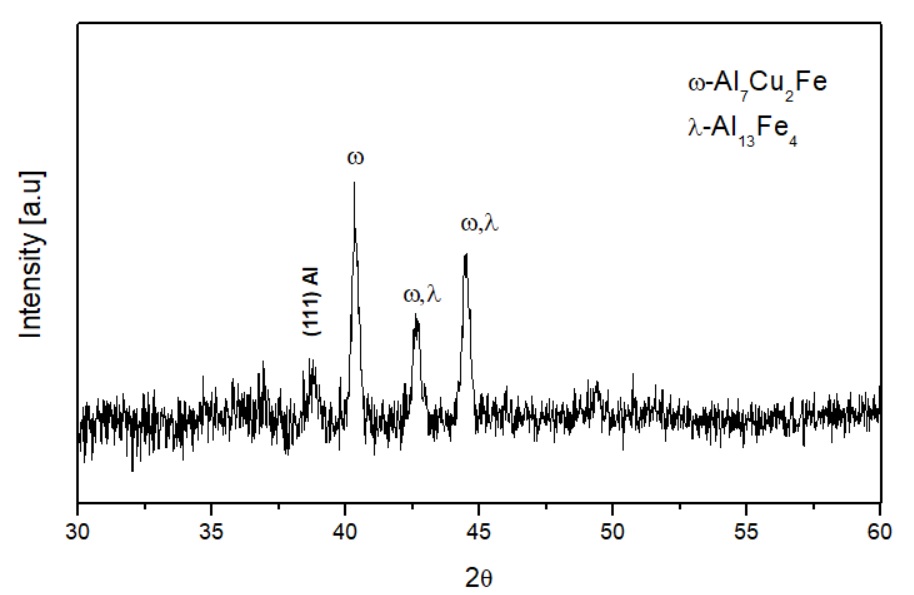


Figure 12 Phase regime: black.


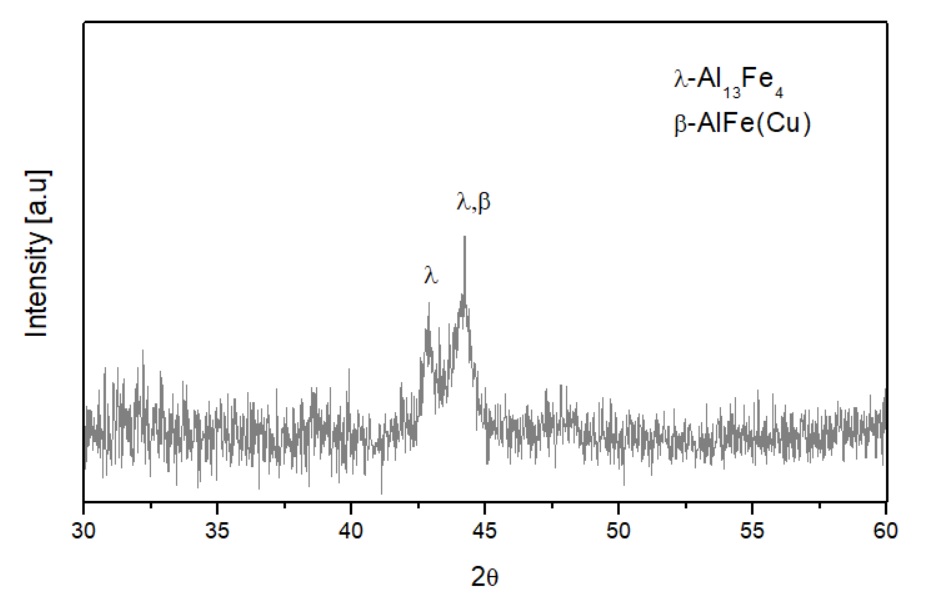


Figure 13 Phase regime: gray.


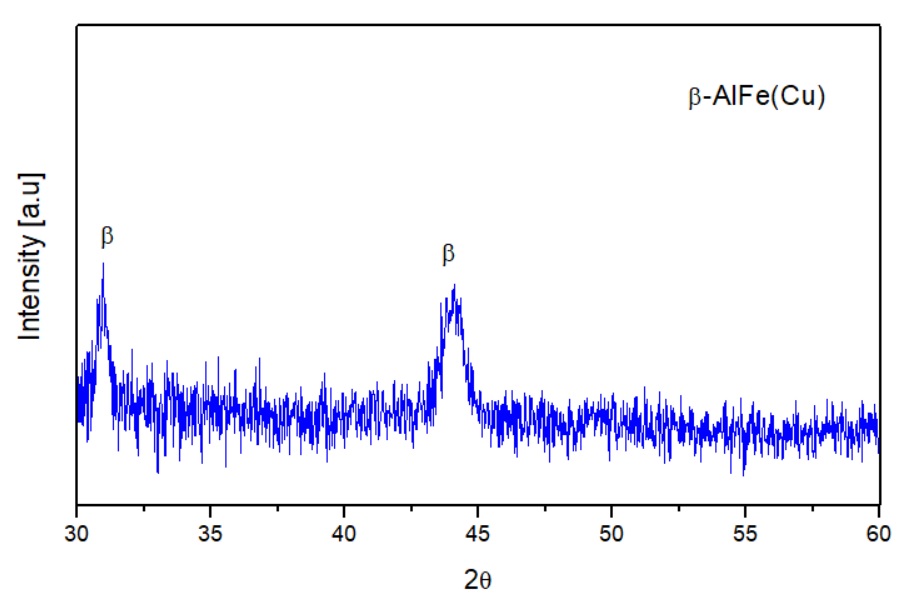


Figure 14 Phase regime: blue.

**XRD patterns – annealing at 600 °C.**





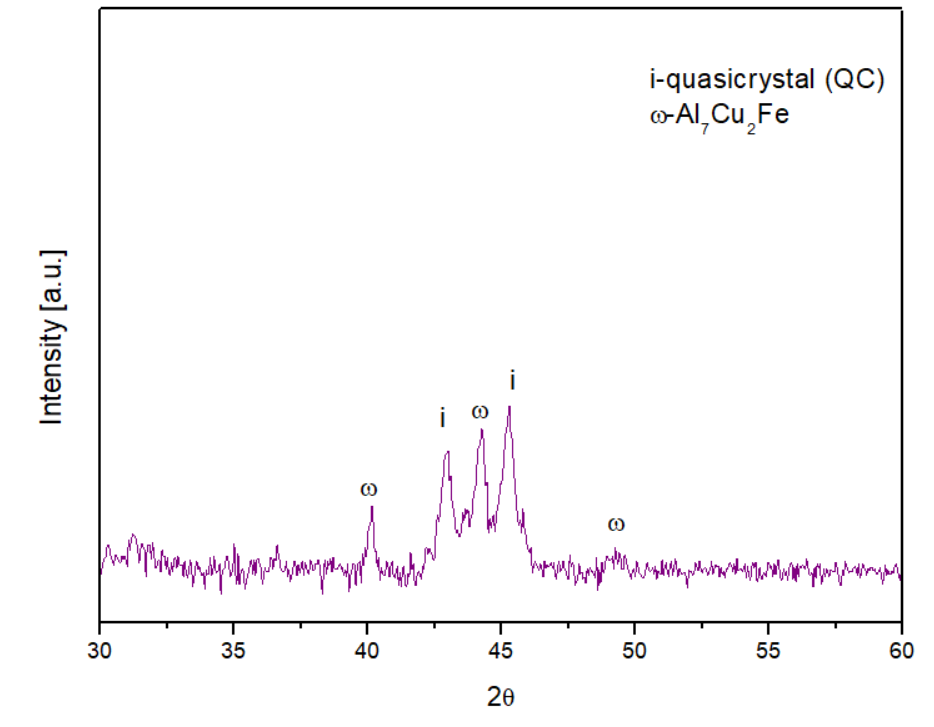


Figure 15 Phase regime: violet.


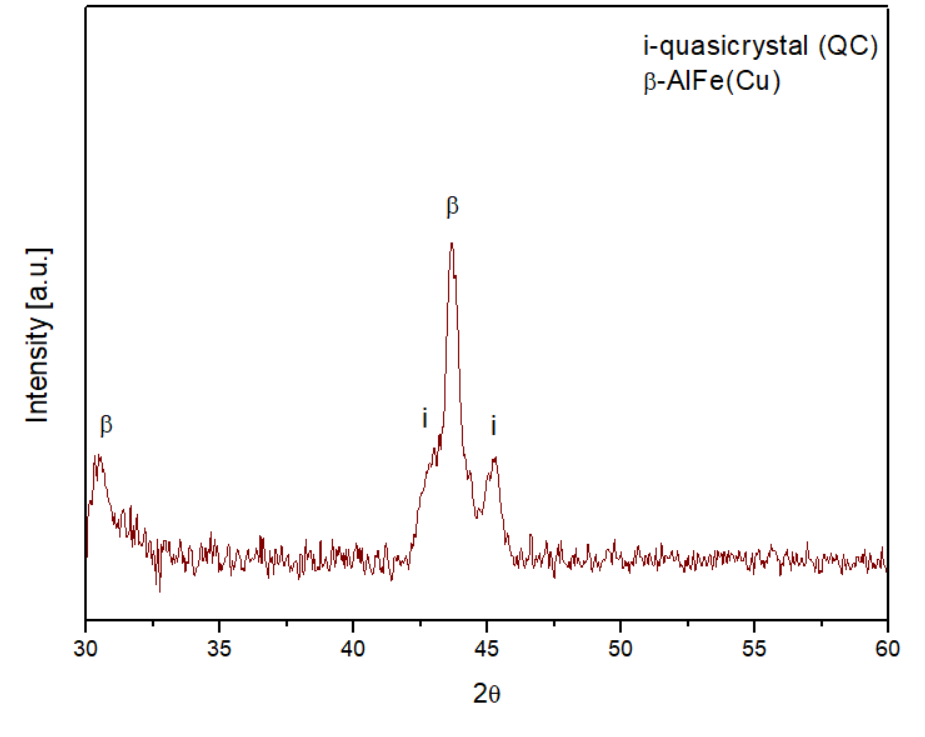


Figure 16 Phase regime: wine.


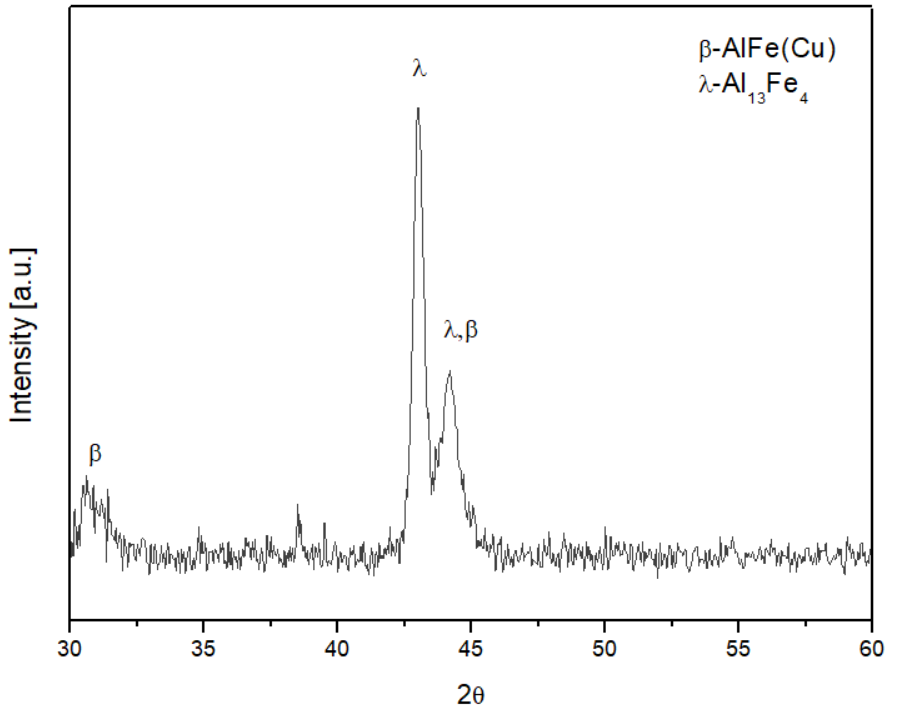


Figure 17 Phase regime: gray.


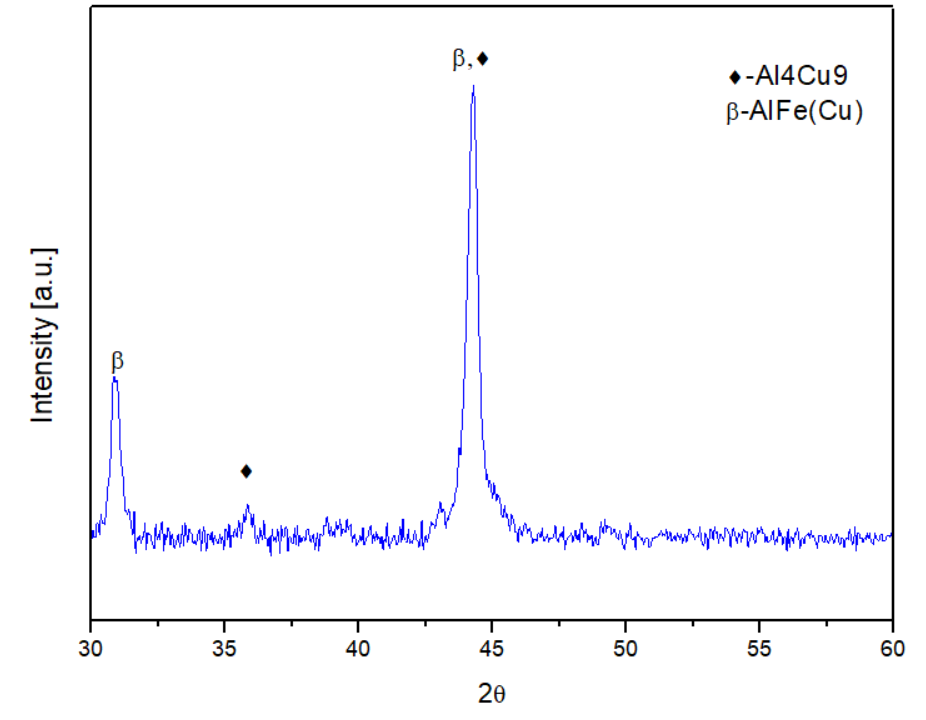


Figure 18 Phase regime: blue.


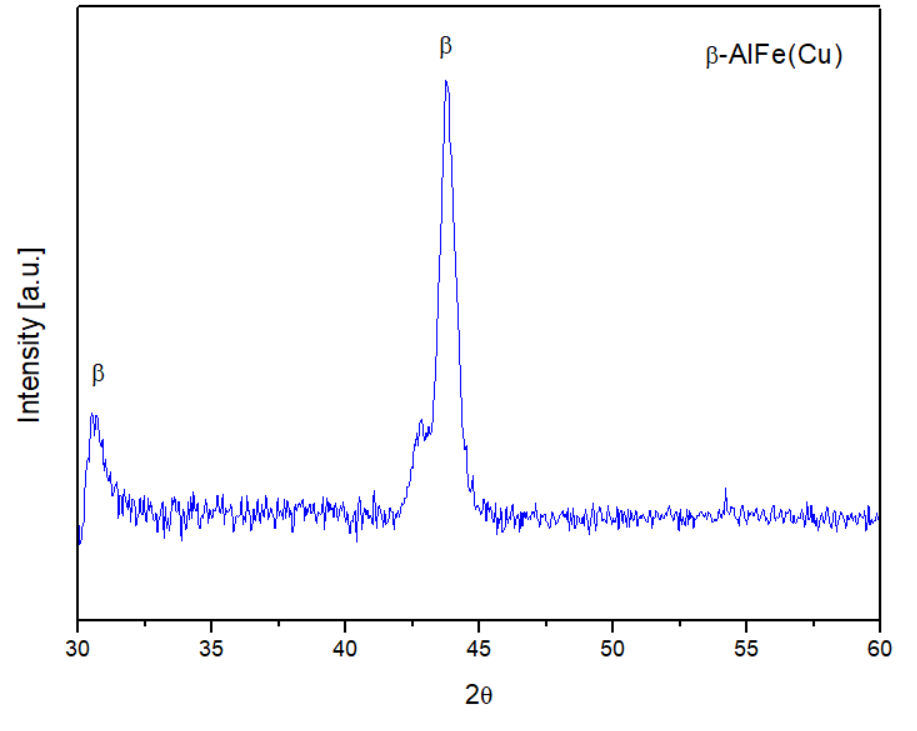


Figure 19 Phase regime: blue.
